# Host Range and Loop-Mediated Isothermal Amplification Detection of *Globisporangium sylvaticum* from Guizhou, China

**DOI:** 10.3390/jof9070752

**Published:** 2023-07-15

**Authors:** Jing Zhang, Xiaonan Sun, Ningjing Ao, Huayan Zou, Huijuan Shao, Koji Kageyama, Wenzhuo Feng

**Affiliations:** 1 Key Laboratory of Agricultural Microbiology, College of Agriculture, Guizhou University, Guiyang 550025, China; 2 College of Resources and Environment, Shandong Agricultural University, Tai’an 271000, China; 3 River Basin Research Center, Gifu University, 1-1 Yanagido, Gifu 501–1193, Japan

**Keywords:** host-range, loop-mediated isothermal amplification detection, *Globisporangium sylvaticum*, cash crop

## Abstract

*Globisporangium*, especially *G. sylvaticum*, causes devastating root rot, blight, and other diseases in various species of cash crops. To investigate the distribution and host range of *G. sylvaticum* in Guizhou, a suitable habitat for this pathogen, we collected 156 root-diseased samples, isolated the pathogens, and found that *G. sylvaticum* is widespread and has eleven host plants, including four novel hosts. Furthermore, to effectively identify *G. sylvaticum*, we developed a simple and dependable method based on loop-mediated isothermal amplification (LAMP), which used a primer set designed from the internal transcribed spacer sequences with high specificity and sensitivity of 1 pg/μL. Additionally, to perform field identification, we used the “Plant-LAMP” method with crude DNA extraction to detect the pathogen in 45 root samples from nine species of plants. Our results showed that this method could effectively detect *G. sylvaticum* in diseased roots. Therefore, our findings not only enrich existing research on the diversity of pathogenic *Globisporangium* in Guizhou but also present an efficient LAMP field detection method that could significantly contribute to plant disease management and prevention.

## 1. Introduction

The genus *Globisporangium* is a recently described taxa that was segregated from *Pythium* [1,2]. *Globisporangium* is an important soil-borne and wide-host range plant pathogen distributed worldwide, including in the crop-growing regions of East Asia, Western Europe, North America, and Australia [3]. During hot and rainy seasons, *Globisporangium* oospores germinate readily and produce many sporangia and zoospores, which spread quickly through water or strong winds, resulting in various diseases [4]. Guizhou is a suitable environment for *Globisporangium* owing to its warm and humid climate, diverse and abundant vegetation, and multiple river systems [5]. Simple water conservation infrastructure and insufficient irrigation and drainage capacities are characteristic of the region’s production base. Root rot and wilt diseases in vegetables and other vital crops occur frequently and spread quickly during seasons of constant rainfall and hot temperatures [6]. Several pathogenic *Globisporangium* species have been identified to be associated with root rot or damping-off of vegetables such as cabbage, ginger, green onions, and lettuce, with *G. sylvaticum* being especially prevalent.

*Globisporangium sylvaticum* is a heterothallic species originally isolated from soil in the United States and later discovered in China, Canada, Turkey, Germany, the Netherlands, and other countries [7,8,9]. Its pathogenicity and toxicity have since been examined in more detail. In Iowa and the Midwestern United States, the pathogen has already caused a large outbreak of soybean seed rot [10]. Globally, a large number of host plants have been identified. It has been associated with maize root rot in Northeast China, lettuce root rot in Italy, base stem rot in Miscanthus in Illinois, and potato tuber rot in North America [11,12,13,14]. Therefore, there is an urgent need to investigate locally infected plants systematically and to develop an efficient diagnostic method to quickly identify this disease and meet the prerequisites for green prevention and control.

Traditionally, selective media containing antinematode and bacterial and fungal agents were used for isolation of *Globisporangium*, followed by further identification of the isolated species based on morphological and other biological characteristics [15]. These features can only be reliably identified by skilled taxonomists and, thus, cannot be reported by farm labourers. In addition, it is difficult to isolate slow-growing pathogens in complex microbial environments and to distinguish the pathogenicity of the isolates. Over the past few decades, molecular techniques for the effective detection of bacteria, oomycetes, and other microorganisms have been widely developed [16,17]. Conventional and real-time polymerase chain reaction (PCR) are the most commonly used methods. Lou and Zhang (2004) used PCR primers from the internal transcribed spacer (ITS) region to specifically detect *G. sylvaticum* [18]. However, PCR testing is time-consuming, requires specialized equipment and is difficult to use for field diagnoses [19]. To overcome these obstacles, many isothermal DNA amplification methods, such as loop-mediated isothermal amplification (LAMP), have been developed to diagnose plant pathogens in situ rapidly [20,21].

Since the development of the LAMP method in 2000, it has been widely applied for the detection of various pathogens and has proven to be a rapid, simple, and efficient method with excellent practical value [22,23,24]. The ingenious design of the dumbbell-shaped DNA does not require thermal denaturation during the reaction and enables rapid, continuous, and specific amplification at a low cost. The by-products of the LAMP process, such as magnesium pyrophosphate, cause the reaction mixture to become more turbid, making it possible to observe the amplification results with the naked eye [22]. Other techniques based on colour changes using dyes have also been used effectively in LAMP assays. These dyes include neutral red (related to pH), HNB (related to Mg^2+^ concentration), and SYBR Green I (related to DNA content) [25,26,27]. Therefore, we believe these techniques will be helpful for the on-site diagnosis of *G. sylvaticum*.

The purpose of this study was to investigate the hosts and distribution features of *G. sylvaticum* in Guizhou, design specific and sensitive LAMP primers for pathogen detection, and develop a simple and reliable method for pathogen detection in each host.

## 2. Materials and Methods

### 2.1. Chemicals

All the primers were synthesized from Sangon Biotech (Shanghai, China). LAMP fluorescent dye and *Bst* 2.0 WarmStart^®^ DNA Polymerase were purchased from New England BioLabs (Beijing, China). The TaKaRa Ex Taq kit was purchased from Takara Bio, Inc. (Beijing, China). The V8 juice was obtained from Campbell Soup Co. (Camden, NJ, USA). The PrepMan Ultra Reagent was obtained from Applied Biosystems (Foster City, CA, USA). All other compounds were obtained from Sangon Biotech (Shanghai, China).

### 2.2. Collection, Isolation, and Conservation

Samples of root or stem rot, wilt, and damping-off from typical field and facility crops, including a variety of grains, vegetables, and flowers, were obtained from various districts of Guizhou between 2021 and 2022. The isolates were obtained by incubating the disease samples at 20 °C for 1–2 days in selective V8 juice agar (V8A) medium (15% clarified V8 juice with 2.5 g/L CaCO_3_ and 2% agar) with nystatin, ampicillin, rifampicin, and miconazole (NARM) [28] and then purifying them using single hyphal or colony tip culture methods. All isolates were conserved on cornmeal agar at the Guizhou University Culture Collection at 20 °C in the dark.

### 2.3. Morphological Studies

*Globisporangium sylvaticum* is heterothallic; therefore, two putative isolates with opposite mating types were incubated in the V8A medium for confrontation culture. The colony and sexual and asexual structures were observed under a microscope after 1–2 weeks of culture at 25 °C in the dark. A minimum of 20 measurements were randomly obtained for each structure using a light microscope. The isolates were morphologically identified based on the diagnostic keys in the “Monograph of the genus *Pythium*” edited by van der Plaats-Niterink (1981) [29].

### 2.4. DNA Extraction, PCR Analysis, and Multi-Locus Phylogeny

PrepMan Ultra Reagent was used to extract genomic DNA of all isolates from mycelia, as described by Baten et al. (2014) [30]. The DNA concentration was measured using a Nanodrop spectrophotometer (Thermo™ Fisher Scientific, Wilmington, DE, USA), and the DNA was diluted to 100 pg/μL for further use. Sequences of the ITS and mitochondrial cytochrome c oxidase subunit 1 (*cox*1) gene regions were used for identification at the species level using the primer sets listed in Table S1 [31,32]. The PCR mixtures (total volume of 25 μL) were prepared using the TaKaRa Ex μTaq kit, and amplification was performed using a PCR device (846-x-070-723, Analytik Jena, Gottingen, Germany), as described in Table S1. Amplification products were separated using a 2% agarose gel (containing nucleic acid dye) and photographed under UV light. PCR products were sequenced by Sangon Biotech (Shanghai, China). The raw sequences were obtained and submitted to GenBank (Table 1). The phylogenetic trees were generated using the Maximum Likelihood (ML) and Maximum Parsimony (MP) methods on the CIPRES web portal (https://www.phylo.org/portal2/login!input.action, accessed on 6 July 2023), utilizing the combined ITS and *cox1* dataset. *Elongisporangium dimorphum* and *E. prolatum* were selected as outgroup. For the ML analysis, the RAxML-HPC BlackBox tool was employed with its default settings. On the other hand, the MP analysis was conducted using the PAUP 4.a168 tool on XSEDE.

### 2.5. Koch’s Postulates

Pure *G. sylvaticum* isolates were cultured in conical flasks containing 100 mL of 10% V8 liquid medium and 50 autoclaved wheat seeds. The flasks were incubated at 150 rpm in the dark at 25 °C for 7 days. Budding wheat seedlings were transplanted into 0.4-L pots with sterilized substrate soil saturated with deionized water. The infection treatment involved placing eight infected wheat seeds near the roots of the seedlings, whereas the control treatment involved placing eight autoclaved seeds. Each group was comprised of five seedlings. All seedlings were planted in the artificial climate chamber (QHS-Z4Z), with a relative humidity of approximately 75% at 25 °C. After 28 days, the occurrence of the disease was observed and recorded, and the diseased roots were placed in the NARM medium.

### 2.6. LAMP and PCR Primer Design

The ITS region, which effectively distinguished *G. sylvaticum* from other species, except *Pythium terrestris* (*G. terrestre*), was selected as the target sequence for designing LAMP and PCR primers. Multiple alignments of different ITS sequences from *G. sylvaticum* and other *Globisporangium* species were analysed using the BioEdit Sequence Alignment Editor software (Figure S1). Regions specific to *G. sylvaticum* were identified and used in primer design. In particular, the unique sequences were located at the 5′ ends of LAMP primers-FIP (or F1c) and BIP (or B1c) and 3′ ends of PCR primers. In addition, two-loop primers were added to accelerate the LAMP reaction. All LAMP and PCR primers were designed and analysed using PrimerExplorer V5 software (https://prime rexplorer.jp, accessed on 28 August 2022) or Primer3web (https://bioinfo.ut.ee/primer3/, accessed on 28 August 2022).

### 2.7. LAMP Reaction

Each LAMP reaction was carried out in a 15 μL reaction mixture containing 1x reaction buffer [20 mM Tris-HCl (pH 8.8), 10 mM KCl, 0.1% (*v*/*v*) Tween 20, 0.8 M betaine, 8 mM MgSO_4_, 10 mM (NH_4_)_2_SO_4_, and 1.4 mM dNTPs], primer mixture (0.2 μM F3 and B3 primers, 0.1 μM F-loop primer, and 1.6 μM FIP and BIP primers), 4.8 U of the *Bst* 2.0 DNA polymerase and template DNA (100 pg for specificity tests), and 0.2 μL of LAMP fluorescent dye. Specificity and sensitivity tests were performed to identify optimal primers. Furthermore, the reaction temperatures of 60, 62.5, 65, and 67.5 °C were tested as primary parameters to optimise the LAMP reaction. Reactions were conducted for 60 min in a 7500 Fast thermocycler (Applied Biosystems), and the real-time fluorescence intensities were recorded at intervals of 30 s, under a constant temperature of 65 °C. To visually evaluate the reaction results, the colour change of SYBR Green I dye was observed. Two drops of mineral oil were added to seal the reaction mixture before initiating the reaction. After the reaction, 2 μL of SYBR Green I (1000×) was added to the tube wall, sealed, and vortexed. Sterile distilled water was employed as a negative control, and genomic DNA from *G. sylvaticum* served as a positive control.

### 2.8. Specificity and Sensitivity of LAMP or PCR Primers in Detecting G. sylvaticum

All primer sets were first checked for specificity using 100 pg of genomic DNA template from two isolates of *G. sylvaticum* (GZso05 and GZco02) and seven genetically related isolates of the *Globisporangium*. One LAMP or PCR primer set with preliminarily determined specificity was further examined in a wide range of species. In total, 42 isolates comprising 25 *Globisporangium*, *9 Pythium*, 2 *Phytopythium*, 3 *Phytophthora* isolates, and 3 isolates of other typical fungal pathogens were studied (Table 2). In addition, a series of 10-fold dilutions from 1 ng/μL to 1 fg/μL of *G. sylvaticum* (GZst02) genomic DNA were used for the sensitivity assay. The LAMP or PCR assay was performed using appropriate primers and reaction conditions as previously described.

### 2.9. Detection of G. sylvaticum in Plant Roots

The “plant LAMP (P-LAMP)” procedure, described by Feng et al. (2015), has been utilized to detect pathogens in various plant roots [33]. Rotting or browned roots were cut into 5 × 1 cm pieces, collected in a 1.5-mL tube, mixed with 100 μL of sterile distilled water, and vortexed for 60 s. The supernatant (1 μL) contained the template DNA for the LAMP reaction. In addition, the root samples were placed on NARM medium and cultivated for 1–3 days at 25 °C. The mycelia grown were transferred to V8 medium or slants for identification based on their morphological and molecular characteristics.

## 3. Results

### 3.1. Identification of G. sylvaticum

**Figure 1:**
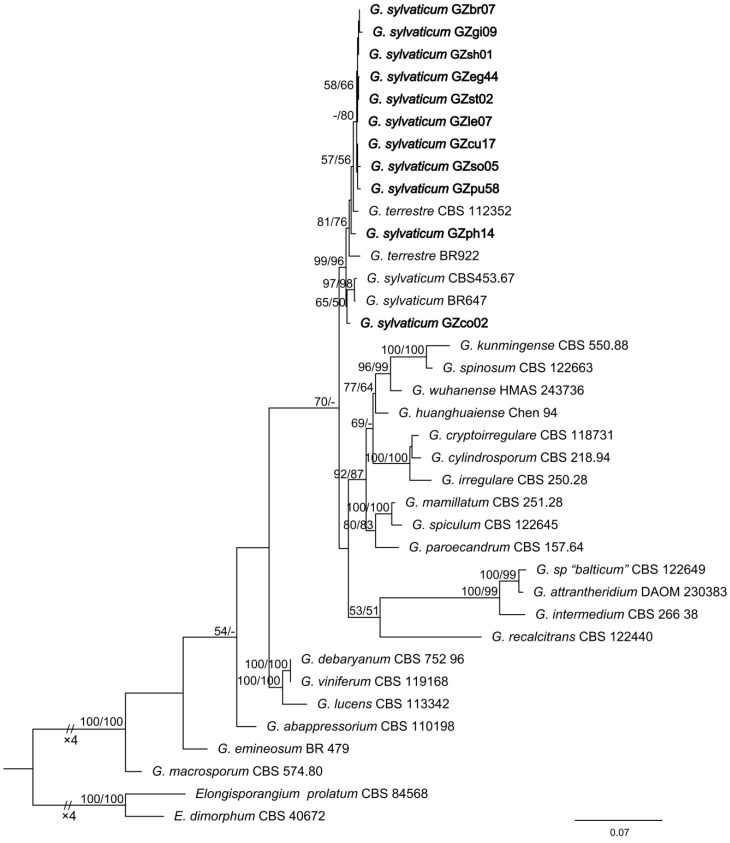
Phylogenetic tree of *Globisporangium sylvaticum* and related species generated using Maximum Likelihood (ML), based on internal transcribed spacer (ITS) and *cox1* sequences. Branch lengths were estimated with RAxML under ML. Numbers on the branches represent bootstrap values (BVs) greater than 50%. ML BVs from RAxML (left) and Maximum parsimony (MP) BVs from PAUP* 4.0a software (right) are shown here.

In total, 156 root-disease samples from 38 plant types were collected from 17 counties in seven cities in Guizhou. The samples were cultured in a selective medium, and 161 isolates of *Globisporangium* were obtained. All isolates were initially identified via ITS sequencing, of which 29 showed 99.5–100% similarity to *G. sylvaticum*. Furthermore, 11 of the 29 isolates were obtained from different plants (Table 2) and further identified using the *cox1* sequence. A phylogenetic tree of the two sequences was constructed. We observed that the 11 isolates exhibited minimal association with the phylogenetic cluster containing *G. sylvaticum* CBS 543.67, *G. sylvaticum* BR647, *G. terrestre* (*P. terrestris*) CBS 112,352, and *G. terrestre* BR922 (Figure 1, Table 1).

**Figure 2:**
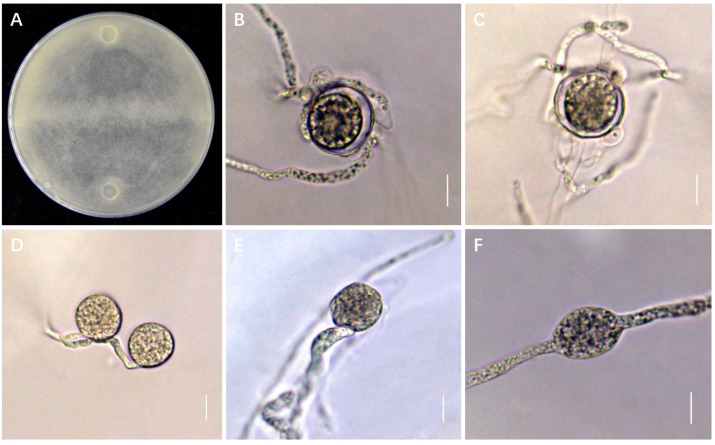
Mating reactions and organ structure in crosses between selected isolates of the *Globisporangium sylvaticum* species. (**A**) GZsh01 × GZco02 mating; (**B**) terminal sporangium and oospore; (**C**) intercalary sporangium and oospore; (**D**–**F**) hyphal swellings: terminal or intercalary; scale bars: 10 µm.

Furthermore, all 11 isolates were heterothallic and were grown in cultures paired with opposite mating-type isolates before morphological identification. The morphologies of GZsh01 × GZco02 on V8A are shown in Figure 2. Smooth, terminal, or intercalary oogonia were observed with a diameter of 19–20.5 µm (average: 20 µm). Approximately 2–4 antheridia were present per oogonium, which were diclinous. The aplerotic oospores had a diameter of 15–18 µm (average: 16.5 µm) and a wall with a thickness of 1–2 µm. We observed increased hyphal swelling, which was globose, limoniform, intercalary, or terminal (Figure 2). The observed morphology was similar to that of *G. sylvaticum* CBS 234.68×230.68. Therefore, we finally identified the isolated strain as *G. sylvaticum*.

### 3.2. Host Plants of G. sylvaticum in Guizhou, China

**Figure 3:**
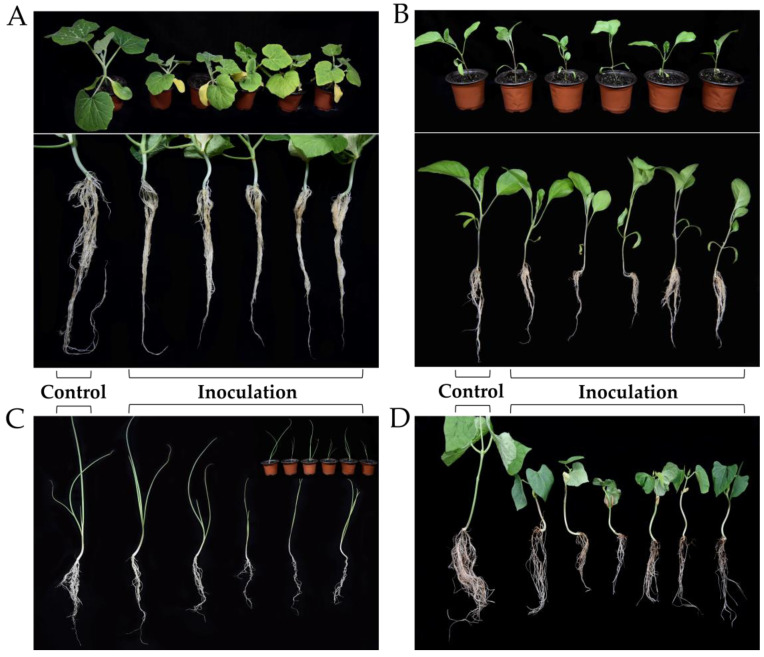
Pathogenicity test results of *Globisporangium sylvaticum* infection of pumpkin (**A**), eggplant (**B**), shallot (**C**), and green bean (**D**).

According to reports, *G. sylvaticum* can infect more than ten different crops, including apples, carrots, lettuce, and soybeans [34,35,36]. The infection of pumpkin, eggplant, spring onions, and green beans by *G. sylvaticum* has not been previously reported; however, the infection of these plants was identified from the plant samples used in this study. A pathogenicity test was performed to determine whether these four plants were hosts of this pathogen. Figure 3 shows the growth results of the inoculated plants, which revealed that among the infected plants, pumpkin, spring onion, and green bean had poor development, and eggplant had slightly poor growth with a decrease in the number of roots and exhibited browning symptoms. Plants in the control group remained symptom-free. The pathogen was reisolated from the diseased roots of all inoculated plants and confirmed to be *G. sylvaticum*.

### 3.3. LAMP and PCR Primer Design and Specificity

Primer sets targeting the ITS region were designed according to the principles of species-specific LAMP and PCR primer design. In total, six LAMP primer sets were designed, each consisting of a modified set on either the F side (F3, FIP) or the B side (B3, BIP) of the primer set. These individual sets were then combined to enhance specificity, and one additional PCR primer set was designed separately.

**Figure 4:**
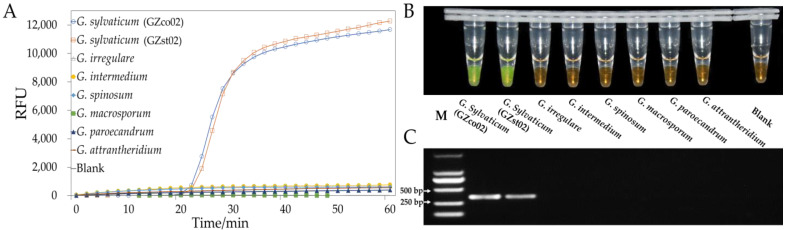
Specificity of the LAMP method. (**A**) LAMP fluorescent dye; (**B**) SYBR Green I; (**C**) PCR of the genomic DNA from *Globisporangium sylvaticum* and seven closely related isolates.

All primer sets were screened using DNA extracts from the eight isolates in *Globisporangium*. The modified set was selected for LAMP because it produced the most specific and consistent amplification results (Figure S1). Two loop primers were designed and added to the selected set (Figure S1). The specificity tests were repeated (Figure 4A,B). PCR primers were also tested (Figure 4C). In addition, the primer sets (Table 3) were further tested with a wide variety of *Globisporangium* and other species and were shown to be highly specific for *G. sylvaticum* (Table 2). Furthermore, a temperature gradient from 60 to 67.5 °C was used to optimize the LAMP reactions for this primer set (Figure S2). An optimal temperature of 65 °C where the reactions exhibited excellent specificity, efficiency, and stability was selected.

### 3.4. Sensitivity of LAMP or PCR In Vitro

**Figure 5:**
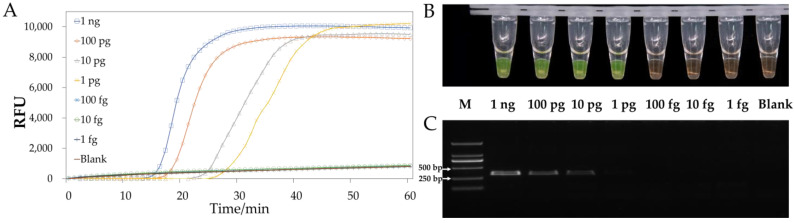
Sensitivity of the LAMP method. (**A**) LAMP fluorescent dye; (**B**) SYBR Green I; (**C**) PCR of serially diluted *Globisporangium sylvaticum* genomic DNA from 1 ng/μL to 1 fg/μL.

Serial dilutions of *G. sylvaticum* (GZst02) genomic DNA were used to evaluate the detection limit of the LAMP method using the selected primer set at the optimal temperature. The sensitivity of the LAMP primers was 1 pg/μL based on real-time fluorescence intensity and SYBR Green I dye analysis, as illustrated in Figure 5A,B. The same dilutions were used for PCR, demonstrating a sensitivity of 10 pg/μL (Figure 5C).

### 3.5. Detection of the Pathogen in Field Samples

**Figure 6:**
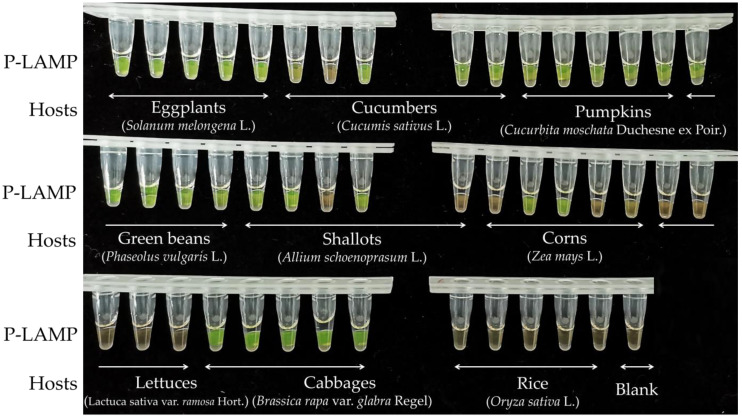
Identification of *Globisporangium sylvaticum* in 45 root samples using P-LAMP. For amplified samples, the dye turns green; for unamplified samples, the dye remains brown.

In total, 45 diseased root samples were obtained from nine plant types (5 of each) in Guizhou (Figure 6). The pathogen was detected using the P-LAMP assay with SYBR Green I dye; brown indicated a negative result, whereas green showed a positive result. *Globisporangium sylvaticum* was found in 29 samples, including 5 eggplant, 4 cucumber, 5 pumpkin, 5 green bean, 3 spring onion, 2 corn, and 5 cabbage samples. The pathogen was not detected in lettuce and rice samples (Figure 6). The isolates were recovered on NARM agar from all positive samples and were confirmed to contain *G. sylvaticum* based on taxonomic characteristics.

## 4. Discussion

We collected several root-diseased samples from important cash crops in different parts of Guizhou, and a total of 13 *Globisporangium* species were found, indicating the substantial distribution potential of *Globisporangium* in Guizhou. Therefore, it is crucial to study prevention and control strategies for crop diseases caused by *Globisporangium*. *Globisporangium sylvaticum* exhibited the highest isolation frequency among the obtained species, indicating that it was the dominating species in the region. Host range and rapid detection methods of this pathogen were thoroughly explored in this study to assist in preventing the pathological development of this pathogen and of the pathogenic *Globisporangium* genus.

Eleven suspected isolates from various plants were identified using molecular and morphological methods. According to the constructed phylogenetic tree, there was no difference in the clustering distance between the isolates and *G. sylvaticum* or *G. terrestre*; furthermore, some sequence differences were found between them, indicating that these pathogens are likely to have relatively rich genetic diversity. Our isolates were eventually identified as *G. sylvaticum* mainly based on heterothallic and morphological characteristics. *Globisporangium terrestre* was first reported by Paul (2002), and the ITS sequence was deposited in GenBank (accession number AY039714) [37]. However, we further “Blast” this sequence and found that it is very different from the now known *G. terrestre* CBS112352 (HQ643857). Robideau et al. (2011) showed that the ITS sequence could not, while *cox1* could, distinguish *G. terrestre* from *G. sylvaticum* in molecular systematics, but both remained in the same cluster [32]. In addition, *G. terrestre* is considered to be homothallic, in contrast to *G. sylvaticum*, but the sexual structures produced by both are similar. Therefore, all strains of these species, including *G. sylvaticum* isolated from Guizhou, demonstrated the need for further taxonomic study.

We discovered various mating types, such as GZsh01 × GZco02, in the region, suggesting that the initial infection sources of the pathogen were probably overwintering oospores. Based on the pathogen analysis or Koch’s postulates between *G. sylvaticum* and the isolated crops, the pathogen is currently known to have eleven hosts, including four novel hosts identified in Guizhou. This vast host range is consistent with the widespread range of hosts that *Globisporangium* is known to inhabit commonly [38]. The results of the pathogenicity experiment showed that the pathogenic ability of the same strain differed among various hosts. *G. sylvaticum* exhibits no host-specificity [39]. However, further studies are required to prove this feature.

Additionally, we investigated the potential of LAMP to detect *G. sylvaticum* using simple DNA extraction. A LAMP primer set including six primers was designed for this pathogen and was confirmed to be specific. Unfortunately, based on the sequence comparison, the primer set was unable to distinguish *G. terrestre*. *Globisporangium terrestre* has only been found in soil as well as soybeans [40]. As a result, the LAMP primers designed here are still practical for *G. sylvaticum* in most host plants. However, highly specific *G. sylvaticum* primers need to be further developed. The reaction was also sensitive, with an acceptable detection limit of 1 pg/μL, which is higher than that of PCR (10 pg/μL). The reaction process was easy to perform and only took 1 h, and the amplification results were successfully measured based on visual observation with colorimetric indicators or real-time fluorescence using a LAMP fluorescent dye. The LAMP products can easily form aerosols, resulting in contamination that is difficult to remove [41]. Thus, to avoid contamination, we added mineral oil to overlay the reaction mixture. Due to the mineral oil overlay, LAMP-amplified products were not recovered for further testing, and electrophoresis was not performed. Furthermore, many researchers have reported that colour change results were consistent with electrophoresis results [42,43]. Therefore, LAMP is an alternative nucleotide amplification method that is rapid, simple, highly sensitive, and suitable for analysing pathogens in the field.

Temperature is the most important factor for a successful reaction and amplification because the pairing ability of primers and template DNA and the efficiency of the amplification enzyme are closely related to temperature. A temperature range of 60–67.5 °C was tested here for optimisation of the reaction. The results showed that *G. sylvaticum* can be specifically and rapidly detected at 65 °C. A higher temperature may increase the specificity of the reaction but reduce the amplification efficiency [44]. To accelerate the amplification, a pair of loop primers was designed and added into the reaction mixture, and the reaction was amplified within 30 min. Although loop primers are not typical primers for LAMP amplification, their addition can significantly improve the reaction rate and shorten reaction time [45].

For the on-site detection of *G. sylvaticum* in the field, we employed the P-LAMP approach. This method employs a crude DNA extraction method that does not require any reagent processing, takes only 1–2 min, and is considered highly efficient for detecting *G. irregulare* or *Phytophthora colocasiae* from plant roots or taro leaves [33,46]. Here, we performed P-LAMP on root samples of 45 plants from nine species and identified the isolates from the tissue cultivated on the NARM medium to verify the detection results. These results demonstrated that the P-LAMP-positive samples contained *G. sylvaticum*. Negative results were observed in 16 samples, particularly in lettuce and rice. These results may be attributed to the potential infection of the symptomatic seedlings by fungi or other oomycetes [47,48,49] or the possibility of inadequate detection of root samples. The former suggests the necessity of implementing a comprehensive plant disease diagnosis system that effectively encompasses all major pathogens of the plant. The latter emphasizes the need to conduct multiple tests on negative samples in order to ensure full verification. In summary, P-LAMP can be used to efficiently detect *G. sylvaticum* in plant roots because of its simplicity, reliability, rapidity, and low cost.

## 5. Conclusions

In this study, we investigated the distribution of *Globisporangium*, especially *G. sylvaticum*, among important cash crops in various regions of Guizhou. Isolates were identified, and their hosts were analysed. *G. sylvaticum* is common in all regions of the Guizhou and has eleven hosts, including four novel hosts identified here. Hence, further research on the diversity of the pathogenic *Globisporangium* genera in Guizhou would have important scientific value. In addition, we designed a LAMP primer set with a specificity and sensitivity of 1 pg/μL to detect *G. sylvaticum* and demonstrated that P-LAMP has the potential to detect pathogens in agricultural fields and may provide a significant contribution to management and prevention, even during the early onset of disease in the field.

## Figures and Tables

**Table 1:** A list of species, isolates, and GenBank accession numbers of sequences used in this study.

Species Name	Isolates	Locality	GenBank Accession No.	
			ITS	COI
*Globisporangium abappressorium*	CBS 110198	USA	HQ643408	HQ708455
*G. cryptoirregulare*	CBS 118731	USA	HQ643515	HQ708561
*G. cylindrosporum*	CBS 218.94	Germany	HQ643516	HQ708562
*G. debaryanum*	CBS 752.96	UK	HQ643519	HQ708565
*G. emineosum*	BR 479	UK	GQ244427	GQ244423
*G. irregulare*	CBS 250.28	Netherlands	HQ643596	HQ708640
*G. lucens*	CBS 113342	UK	HQ643681	HQ708725
*G. mamillatum*	CBS 251.28	Netherlands	HQ643687	HQ708731
*G. paroecandrum*	CBS 157.64	Australia	HQ643731	HQ708772
*G. recalcitrans*	CBS 122440	Spain	DQ357833	EF426549
*G. spiculum*	CBS 122645	France	HQ643790	HQ708831
*G. spinosum*	CBS 122663	India	HQ643791	HQ708832
*G. sylvaticum*	CBS 453.67	USA	HQ643845	HQ708886
*G. sylvaticum*	BR647	Netherlands	HQ643847	HQ708888
*G. terrestre*	CBS 112352	France	HQ643857	HQ708898
*G. terrestre*	BR922	USA	HQ643856	HQ708897
*G. viniferum*	CBS 119168	France	HQ643956	HQ708997
*G. sylvaticum*	GZsh01	China	OQ654058	OQ694389
*G. sylvaticum*	GZph14	China	OQ654059	OQ694390
*G. sylvaticum*	GZco02	China	OQ654060	OQ694391
*G. sylvaticum*	GZst02	China	OQ654061	OQ694392
*G. sylvaticum*	GZso05	China	OQ654062	OQ694393
*G. sylvaticum*	GZle07	China	OQ654063	OQ694394
*G. sylvaticum*	GZbr07	China	OQ654064	OQ694395
*G. sylvaticum*	GZpu58	China	OQ654065	OQ694396
*G. sylvaticum*	GZcu17	China	OQ654066	OQ694397
*G. sylvaticum*	GZgi09	China	OQ654067	OQ694398
*G. sylvaticum*	GZeg44	China	OQ654068	OQ694399
*Elongisporangium dimorphum*	CBS 40672	USA	HQ643525	HQ708571
*E. prolatum*	CBS 84568	USA	HQ643754	HQ708795

**Table 2:** Isolates used in this study for specificity testing of the LAMP and PCR primers.

Species	Clade	Isolates ^a^	Origin	Detection	
				LAMP	PCR
*Pythium aphanidermatum*	A	GZHca2	*Capsicum annuum* L.	−	−
*P. giumdeliense*	A	GZHs21	Soil	−	−
*P. aristosporum*	B	GZWco5	Corns (*Zea mays* L.)	−	−
*P. aquatile*	B	GZal1	*Allium tuberosum* Rottler ex Sprengle	−	−
*P. deliense*	B	GZAbr6	*Brassica rapa* var. *glabra* Regel	−	−
*P. dissotocum*	B	GZbr24	*Brassica rapa* var. *glabra* Regel	−	−
*P. inflatum*	B	GZHs55	Soil	−	−
*P. torulosum*	B	GZHs12	Soil	−	−
*P. oligandrum*	D	GZHs172	Soil	−	−
*Globisporangium hypogynum*	E	GZbr2	*Brassica napus* L.	−	−
*G. middletonii*	E	GZHs43	Water	−	−
*G. attrantheridium*	F	GZLra1	*Raphanus sativus* L.	−	−
*G. intermedium*	F	GZbr1	*Brassica rapa* var. *chinensis* (Linnaeus) Kitamura	−	−
*G. irregulare*	F	GZvi11	Vigna unguiculata (Linn.) Walp.	−	−
*G. irregulare*	F	GZLca2	*Capsicum annuum* L.	−	−
*G. macrosporum*	F	GZHZgl3	*Glycine max* (Linn.) Merr.	−	−
*G. paroecandrum*	F	GZHco1	*Coriandrum sativum* L.	−	−
*G. spinosum*	F	GZbc1	*Brassica chinensis* L.	−	−
*G. spinosum*	F	GZvi1	*Vicia faba* L.	−	−
*G. sylvaticum*	F	GZsh01	Shallots (*Allium schoenoprasum* L.)	+	+
*G. sylvaticum*	F	GZph14	Green beans (*Phaseolus vulgaris* L.)	+	+
*G. sylvaticum*	F	GZco02	Corns (*Zea mays* L.)	+	+
*G. sylvaticum*	F	GZst02	Strawberry (*Fragaria*× *ananassa* Duch.)	+	+
*G. sylvaticum*	F	GZso05	Soybean (*Glycine max* (L.) Merr.)	+	+
*G. sylvaticum*	F	GZle07	Lettuces (*Lactuca sativa* var. *ramosa* Hort.)	+	+
*G. sylvaticum*	F	GZbr07	*Brassica napus* L.	+	+
*G. sylvaticum*	F	GZpu58	Pumpkins (*Cucurbita moschata* Duchesne ex Poir.)	+	+
*G. sylvaticum*	F	GZcu17	Cucumbers (*Cucumis sativus* L.)	+	+
*G. sylvaticum*	F	GZgi09	Ginger (*Zingiber officinale* Roscoe)	+	+
*G. sylvaticum*	F	GZeg44	Eggplants (*Solanum melongena* L.)	+	+
*G. parvum*	G	GZal2	*Allium schoenoprasum* L.	−	−
*G. heterothallicum*	I	Gzla21	*Lactuca*	−	−
*G. ultimum*	I	GZph1	*Phaseolus vulgaris Linn.*	−	−
*G. nodosum*	J	GZHs15	Soil	−	−
*Phytopythium helicoides*	K	GZw1	Water	−	−
*Phy. vexans*	K	GZHs24	Soil	−	−
*Phytophthora nicotianae*	1	GZst31	Strawberry (Fragaria× ananassa Duch.)	−	−
*Ph. cactorum*	1	GZst21	Strawberry (Fragaria× ananassa Duch.)	−	−
*Ph. capsic*	2	GZsm21	*Solanum melongena* L.	−	−
*Colletotrichum siamense*		1-0	*Camellia sinensis* (L.) O. Ktze.	−	−
*Alternaria tenuissima*		V832	*Solanum tuberosum* L.	−	−
*Fusarium oxysporum*		1-7	*Capsicum annuum* L.	−	−

^a^ isolates were maintained in the Culture Collection at the Department of Plant Pathology, Agriculture College, Guizhou University, China. LAMP: loop-mediated isothermal amplification; PCR: polymerase chain reaction.

**Table 3:** Primer sets used in this study.

Species	Primer Set	Primers	Sequences (5′-3′)	Region Amplified
*G. sylvaticum*	L-Psy (LAMP)	F3	TGCTTATTGGGTGTCTGTTC	rDNA-ITS
		FIP	AGCCGCCCACTACTAACAA~TCGCCTTGAGGTGTACTGG	
		B3	TCTTGTCTGATATCAGGTCCA	
		BIP	ACTTGTGCAATTGGCAGAA~CAGGATCAAACCCGGAGTAC	
		F-loop	AACCAGTTCAATCCCACAGC	
	P-Psy(PCR)	For	TTCAAACCCCATACCTAACTT	rDNA-ITS
		Rev	CGCAAGTTGTGCATAAACAA	

## Data Availability

The study did not report any data.

## Supplementary Materials

The following supporting information can be downloaded at: https://www.mdpi.com/article/10.3390/jof9070752/s1, Figure S1: Design of LAMP primers specific for *Globisporangium sylvaticum* based on ITS sequences. Nucleotide sequence alignment of ITS sequences from *G. sylvaticum* and closely related isolates. Partial sequences of rDNA-ITS and the location of five LAMP primers [F3, B3, FIP (F1c-F2), BIP (B1c-B2), and F-loop] are shown. Arrows indicate the direction of extension; Figure S2: Specificity of the LAMP reaction at different temperatures: (A) 60 °C, (B) 62.5 °C, (C) 65 °C, and (D) 67.5 °C for 60 min; Table S1: The PCR primers and PCR systems used in this study.
