# Supplementary material for: Host Range and Loop-Mediated Isothermal Amplification Detection of Globisporangium sylvaticum from Guizhou, China
Source: J Fungi (Basel). 2023 Jul 15;9(7):752. doi: 10.3390/jof9070752 (PMC10381608; doi:10.3390/jof9070752)
Supplement: Supplementary file 1 [file jof-09-00752-s001.zip › jof-2478424-supplementary.pdf]

Supplementary Material

Table S1. The PCR primers and PCR systems used in this study.

PCR regions	Primers (5'-3')		PCR mixtures (25 μL)					PCR conditions					References
			Ex Taq buffer	dNTPs	Each primer	Ex Taq polymerase	Bovine serum albumin	Predenaturation	35 cycles			Final extension	
									Denaturation	Annealing	extension		
**ITS**	ITS 5	GGAAGTAAAAGTCGTAACAAGG	1×	0.2 mM	0.2 μM	0.625 U	0.4 mg	94°C for 3 min	94°C for 30 s	55°C for 30 s	72°C for 1 min	72°C for 10 min	[?]
	ITS 4	TCCTCCGCTTATTGATATGC											
***cox*1**	OomCoxI-Levup	TCAWCWMGATGGCTTTTTTCAAC	1×	0.2 mM	0.5 μM	0.625 U	0.4 mg	94°C for 3 min	94°C for 1 min	55°C for 1 min	72°C for 1 min	72°C for 10 min	[?]
	OomCoxI-Levlo	CYTCHGGRTGWCCRAAAAACCAAA											
**Detection**	For	TTCAAACCCCATACCTAACTT	1×	0.2 mM	0.2 μM	0.625 U	0.4 mg	94°C for 3 min	94°C for 30 s	58°C for 30 s	72°C for 1 min	72°C for 10 min	This study
	Rev	CGCAAGTTGTGCATAAACAA											

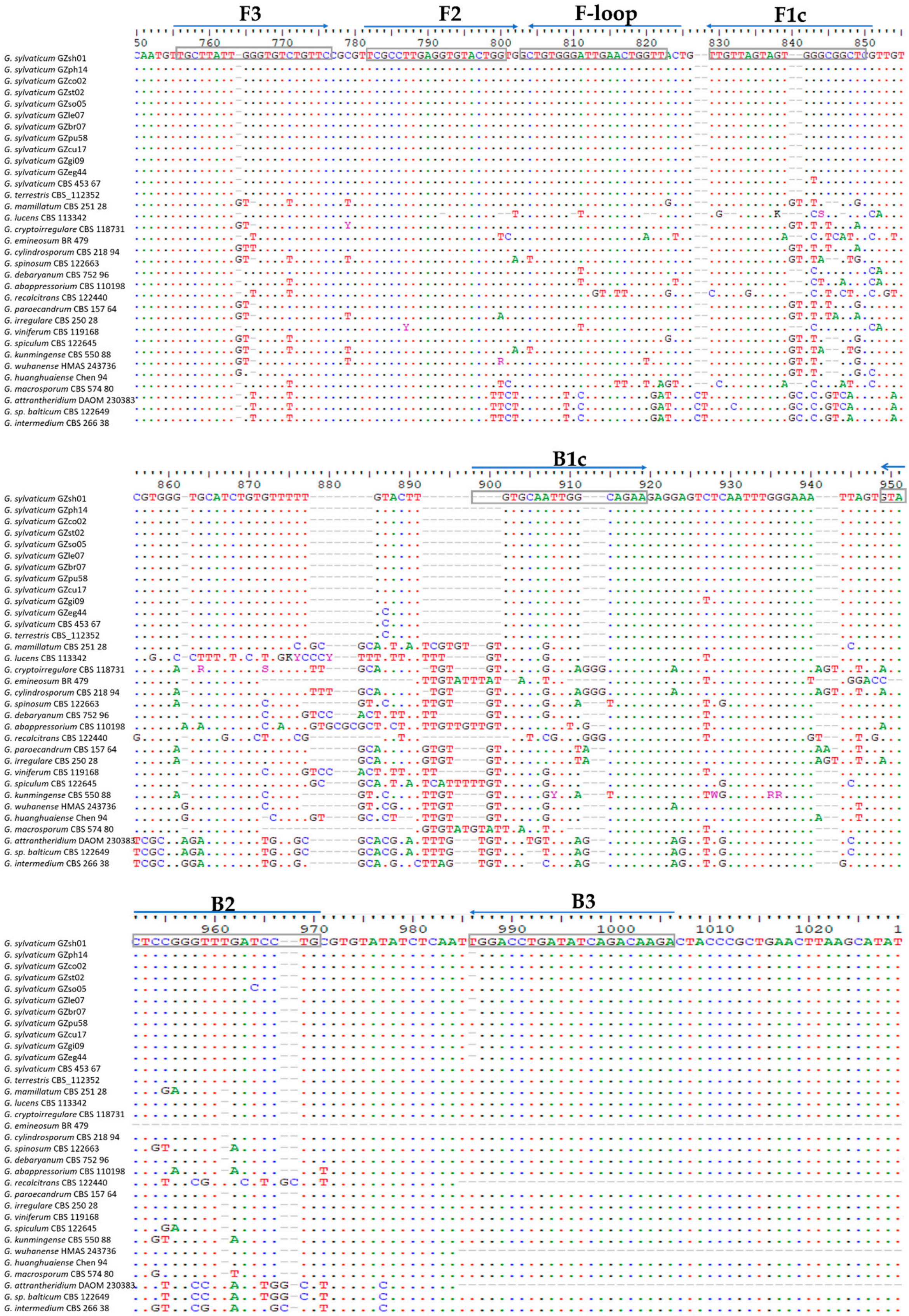

Figure S1. Design of LAMP primers specific for *Globisporangium sylvaticum* based on ITS sequences. Nucleotide sequence alignment of ITS sequences from *G. sylvaticum* and closely related isolates. Partial sequences of ITS and the location of six LAMP primers [F3, B3, FIP (F1c-F2), BIP (B1c-B2), and F-loop] are shown. Arrows indicate the direction of extension.

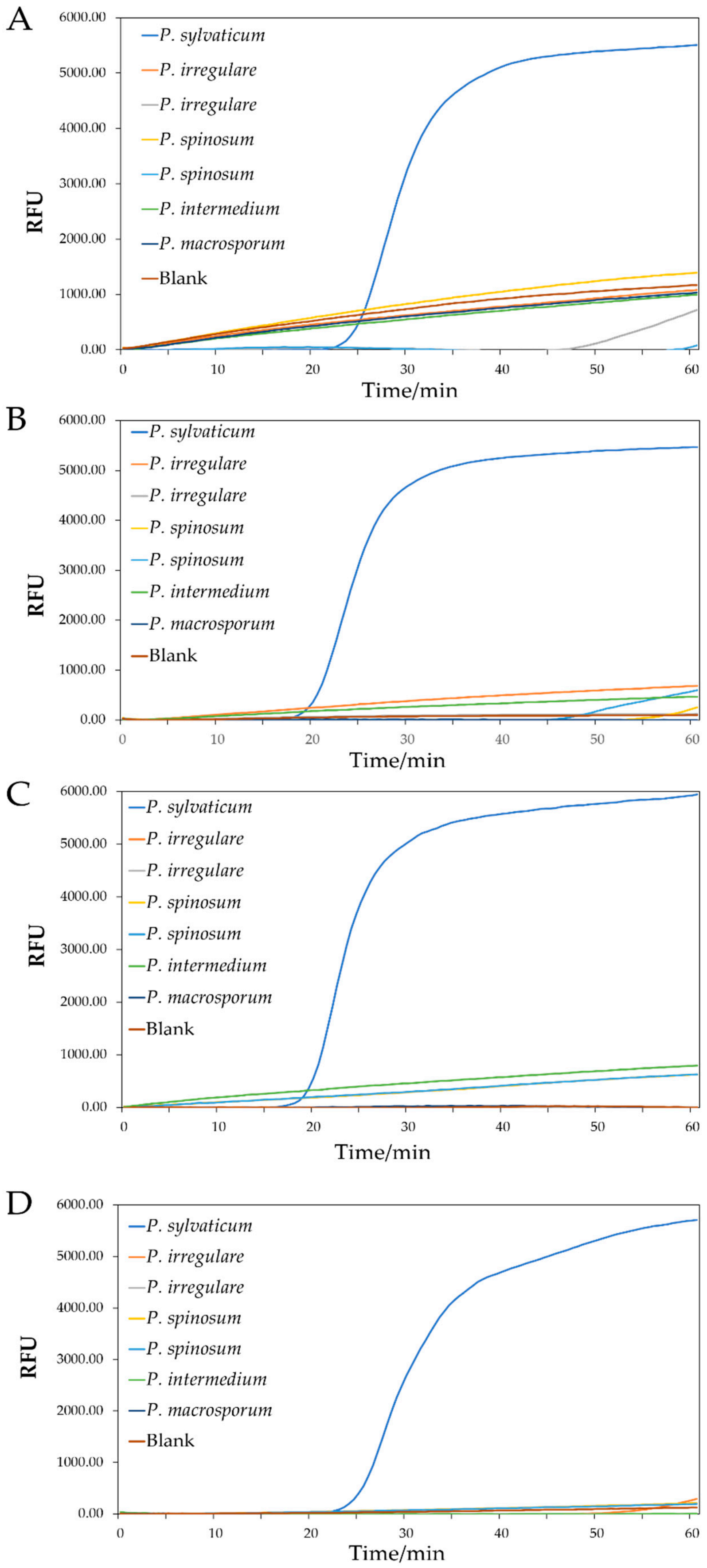

Figure S2. Specificity of the LAMP reaction at different temperatures: (**A**) 60 °C, (**B**) 62.5 °C, (**C**) 65 °C, and (**D**) 67.5 °C for 60 min.
